# Medication Reconciliation in Patients Hospitalized in a Cardiology Unit

**DOI:** 10.1371/journal.pone.0115491

**Published:** 2014-12-22

**Authors:** Gabriella Fernandes Magalhães, Gláucia Beisl Noblat de Carvalho Santos, Mário Borges Rosa, Lúcia de Araújo Costa Beisl Noblat

**Affiliations:** 1 Multidisciplinary Comprehensive Health Residency in adult health care focused on cardiovascular care at Professor Edgard Santos University Hospital, Federal University of Bahia State (UFBA), Salvador, Bahia, Brazil; 2 Professor Edgard Santos University Hospital, Federal University of Bahia State (UFBA), Salvador, Bahia Brazil; 3 Hospital Foundation of Minas Gerais State (FHEMIG); Institute for Safe Medication Practices Brazil, Belo Horizonte, Minas Gerais, Brazil; 4 Faculty of Pharmacy, Federal University of Bahia (UFBA), Salvador, Bahia, Brazil; Professor Edgard Santos University Hospital, Federal University of Bahia State (UFBA), Salvador, Bahia Brazil; University of Padova, Italy

## Abstract

**Objectives:**

To compare drugs prescribed on hospital admission with the list of drugs taken prior to admission for adult patients admitted to a cardiology unit and to identify the role of a pharmacist in identifying and resolving medication discrepancies.

**Method:**

This study was conducted in a 300 bed university hospital in Brazil. Clinical pharmacists taking medication histories and reconciling medications prescribed on admission with a list of drugs used prior to admission. Discrepancies were classified as justified (e.g., based on the pharmacotherapeutic guidelines of the hospital studied) or unintentional. Treatments were reviewed within 48 hours following hospitalization. Unintentional discrepancies were further classified according to the categorization of medication error severity. Pharmacists verbally contacted the prescriber to recommend actions to resolve the discrepancies.

**Results:**

A total of 181 discrepancies were found in 50 patients (86%). Of these discrepancies, 149 (82.3%) were justified changes to the patient's home medication regimen; however, 32 (17.7%) discrepancies found in 24 patients were unintentional. Pharmacists made 31 interventions and 23 (74.2%) were accepted. Among unintentional discrepancies, the most common was a different medication dose on admission (42%). Of the unintentional discrepancies 13 (40.6%) were classified as error without harm, 11 (34.4%) were classified as error without harm but which could affect the patient and require monitoring, 3 (9.4%) as errors could have resulted in harm and 5 (15.6%) were classified as circumstances or events that have the capacity to cause harm.

**Conclusion:**

The results revealed a high number of unintentional discrepancies and the pharmacist can play an important role by intervening and correcting medication errors at a hospital cardiology unit.

## Introduction

Medication errors in hospitals are common and potentially harmful [1], [2]. Care interfaces are vulnerable points for the occurrence of drug-related incidents [3]. Medication reconciliation, is a process proven to reduce errors occurring at these transition points [3]. The process consists of creating a comprehensive and accurate list of all medications used by the patient prior to admission and reconciling this with the medications prescribed on admission [4]. Many types of medication errors, such as the inadvertent omission of necessary medications used before admission, can be prevented by adopting this procedure [4].

Cornish et al found that 81 (53.6%) of the 151 patients included in their 2005 study had, at least, one unintentional medication discrepancy on admission, which suggests that medication errors on admission are common [5]. These authors concluded that medication reconciliation proved to be a powerful strategy to reduce medication errors.

Medication reconciliation is an important strategy to reduce medication error and potential harm [6]. A study conducted by Quélennec et al showed that a combined intervention of pharmacists and physicians in a collaborative medication reconciliation process had a high potential to reduce clinically relevant errors on hospital admission [7]. Medication reconciliation performed by clinical pharmacists increases the safety of patients in the admission process [8].

In 2003, the U.S. Joint Commission for Accreditation of Healthcare Organizations (JCAHO) [9] recognized that errors stemming from lack of medication reconciliation increased the risk of patient harm. Medication reconciliation was then included in their standards, for the first time, as a strategy to improve patient safety.

Between 2006 and 2008, the World Health Organization (WHO) established a Standardized Operating Protocol to prevent medication errors due to incomplete or miscommunicated information during transitions in care [10].

In 2007, the National Institute for Health and Clinical Excellence (NICE) and the National Patient Safety Agency in the U.K. [11] published a solution guide for adult inpatient medication reconciliation. In this document, NICE states that the pharmacist should perform medication reconciliation on hospital admission and that the responsibility of the pharmacist and other staff members should be well defined and may vary among clinical areas.

In Spain, in January 2009, the Catalan Society of Clinical Pharmacy [12] released a guide for the implementation of medication reconciliation programs in Healthcare Centers, with the aim of contributing to the prevention and improvement of the patient care process.

The experiences of medication reconciliation initiatives in Brazil are increasingly being published in congress annals, [13]–[15]. The available published data, although limited, indicate that few pharmacists perform clinical activities in Brazil. A study carried out to identify the extent to which services are provided showed that pharmacotherapy follow-up is rare in the Brazilian hospitals assessed [16]. Delgado et al (2007) [17] mention that this process is an opportunity to improve pharmaceutical care since it requires direct patient contact.

Our study aimed to compare drugs prescribed at hospital admission with the list of drugs taken prior to admission by adult patients admitted to a cardiology unit and to identify the unique role of a pharmacist in identifying and resolving medication discrepancies.

## Material and Methods

This is a research study containing a reconciled list that was prepared with hospital-prescribed medication at admission and home medication used by patients at a cardiology unit of a university hospital. The patient's medications were changed to the list of medications that are readily available in the hospital and according patient's clinical status during the period from November 1, 2012 to March 31, 2013.

This study enrolled patients over the age of 18 admitted to the cardiology unit, who were using at least 3 medicines before admission, had a minimum 24-hour stay in the unit and were either available for interview or accompanied by a family member or a caregiver to provide data.

Patients transferred from other clinical or hospital units or that did not meet the inclusion criteria were excluded from this study.

Following a one month pilot study, data were collected by a clinical pharmacist using the medication reconciliation form adapted from Ketchum (2005) [18]. Treatments were reviewed within the 48-hour period following admission. An interview was conducted with patients or their family members to find out their medication history. Pre-admission medicine use was recorded on the reconciliation form.

Prescriptions received by the pharmacy were compared with the medications listed on the reconciliation form and the patients' medical records to identify whether all drugs used by patients before admission were prescribed on admission. The unintentional discrepancies were discussed with the prescriber and modified, if necessary. All known discrepancies and pharmacist interventions were documented on the reconciliation form. The following data were collected from medical records and during the interview: clinical history, patient's name, age, sex, reason for hospitalization, medication used and prescribed. After gathering the information, data were analyzed and the discrepancies found were classified according to the types proposed by Delgado et al in 2007 [17]: no discrepancy; justified discrepancy and unintentional discrepancy.

*Justified discrepancy*: when the prescription drug is justified by the clinical situation; the medical decision not to prescribe a medication or to change the dose, frequency or route according to protocols; the replacement therapy as per hospital pharmacotherapeutic guides.

*Unintentional discrepancy*: when omission of a required drug occurs; addition of medication not justified by the patient's clinical condition; replacement without clinical justification or reason for product availability; different dose, route of administration, frequency, time and method of administration; duplication; drug-drug interaction; [19].

Symptomatic medications prescribed on admission at the physician's discretion or according to the patients' needs were not considered discrepancies, provided that such medications were not contraindicated for patients included in this study.

Unintentional discrepancies were also classified by their potential to cause harm, according to the classification method proposed by The National Coordinating Council for Medication Error Reporting and Prevention (NCC MERP) and adapted by Gleason et al (2010) [20]. The scale proposed by Gleason et al considers three categories: no potential harm (NCC MERP category C); monitoring or intervention potentially required to preclude harm (NCC MERP category D); potential harm (NCC MERP categories E and above). We considered the discrepancy's potential harm to the patient had reconciliation not been performed within 48 hours.

The drug classes were identified according to the Anatomical Therapeutic Chemical (ATC) classification system.

This work was approved by the Ethics Committee in Research of the University Hospital Professor Edgard Santos. Patients voluntarily gave written informed consent to participate in this study.

Data were entered into Microsoft Excel 2007 spreadsheets and results were analyzed using descriptive statistics.

## Results

**Figure 1:**
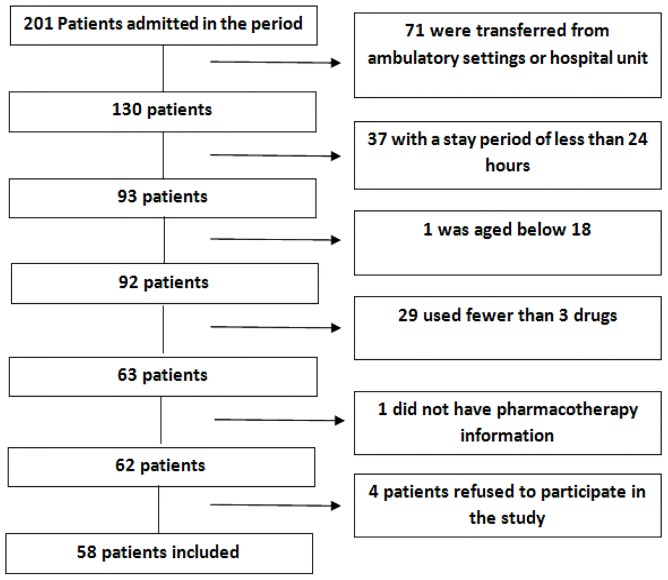
Summary of patient recruitment and enrollment.

During the period of the study, 201 patients were admitted to the cardiology service. Of these, 58 were included in the study as they met the inclusion criteria (Fig. 1). Twenty-four (41.4%) were men and 34 (58.6%) were women; the age average of participants was 65 years. The average number of pre-admission medications was 6.2, with a total of 347 medications. Table 1 describes the features of the patients included in this study.

**Table 1:** Patients' features.

Features	Value
Sex	
Male	41.4%
Female	58.6%
Age, year (average)	65
Reason for hospitalization	
Heart Surgery	13
implantation or replacement of the pacemaker's generator	16
Catheterization	15
Decompensated heart failure	11
Other	3
Source for the medication history information	
Patient	74.1%
Family	25.9%
Number of drugs on admission (average)	6.2

A total of 347 medications were reconciled in 58 patients and 181 discrepancies were found in 50 patients. Of these, 45 (24.9%) discrepancies were related the inclusion of a medication on the admission medication prescription which the patient had not been using prior to admission, due to the patient's needs, 84 (46.4%) were discontinued or had their route, frequency or dose adjusted according to the patient's clinical condition, 20 (11%) were related to substitution to the hospital's formulary medication and 32 (17.7%) were unintentional.

Of the 32 unintentional discrepancies identified in 24 patients, 31 were discussed with the prescribers (Table 2). Only one discrepancy did not require a pharmacist intervention, since the prescriber himself identified and resolved the discrepancy. The clinical pharmacist performed a total of 30 interventions; 22 recommendations (73%) were accepted and 8 (27%) rejected. Most rejected interventions were frequency-related discrepancies, for example, the patient using atenolol 100 mg once a day when 50 milligrams twice daily were prescribed, and prescribers judged it was unnecessary to change. Eight patients showed no discrepancy (Table 3).

**Table 2:** Examples of unintentional discrepancies and interventions performed.

Case Description	Unintentional Discrepancy	Pharmaceutical Intervention
Patient with Congestive Heart Failure	Beta blockers omitted	Prescription requested
Patient with Prostatic Hyperplasia	Finasteride and Doxazosin omitted	Prescription requested
Patient with Chronic Obstructive Pulmonary Disease	Tiotropium/salmeterol+Fluticasone omitted	Prescription requested
Hypertensive Patient	Increased dose of losartan from 100 mg to 150 mg/day	Dose modification discussed
Patient with Heart Failure	Patient was using propranolol, metoprolol prescribed, drug not selected at hospital	Suggested replacement of metoprolol with carvedilol, greater evidence of benefits than propranolol
Patient with Gastritis	Omeprazole omitted	Prescription requested
with isolated hypertriglyceridemia	Simvastatin prescribed instead of Fibrate used by patient	Suggested modification of prescription to bezafibrate, selected drug
Patient with Coronary Artery Disease	Simvastatin omitted	Requested prescription.

**Table 3:** Medication discrepancies per patient.

Frequency of discrepancies	Number of Patients	Total discrepancies
0	8	0
1	6	6
2	10	20
3	10	30
4	8	32
5	10	50
6	2	12
7	2	14
8	1	8
9	1	9
Total	58	181

**Figure 2:**
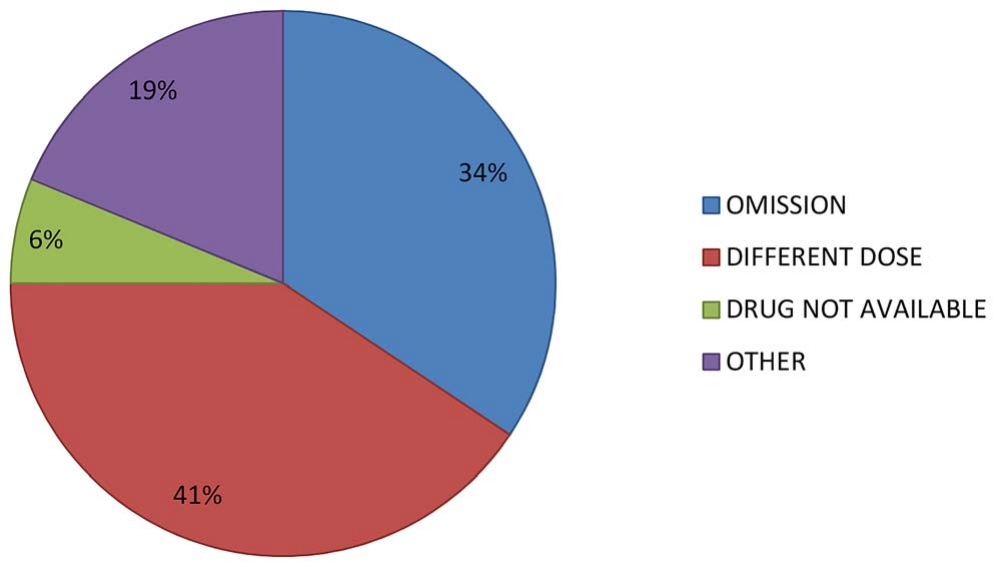
Unintentional discrepancies.

The most common type of unintentional discrepancy was differences in medication doses (41%) followed by omission of medications (34%). (Fig. 2).

Most unintentional discrepancies [n = 13 (40.6%)] were classified in the category error without harm. Eleven (34.4%) unintentional discrepancies were classified in the category error without harm but which could have required patient monitoring or intervention to prevent harm had a pharmacist not intervened (56.2%); for example, increasing the dose of losartan 100 mg/day to 150 mg/day. Three unintentional discrepancies (9.4%) could have resulted in harm to the patient (category E–F) had the pharmacist not intervened; such as the omission of a beta blocker in a patient with congestive heart failure. Five unintentional discrepancies (15.6%) were classified as circumstances or events that have the capacity to cause harm for example, replacing one drug with another of the same pharmacological class (enalapril instead of captopril), without clinical justification. No unintentional discrepancies were judged potentially lethal to the patient.

The drug classes most commonly associated with unintentional discrepancies were identified. Beta blockers and agents acting on the rennin-angiotensin system accounted for 18.8% and 15.6% of records, respectively, in this cardiology population (Table 4).

**Table 4:** Types of medication associated with unintentional discrepancies.

ATC Class *	Frequency of unintentinal discrepancies	%
Organic nitrates	2	6.3
Proton pump inhibitors	2	6.3
Digitalis	2	6,3
Diuretic agents	4	12.5
Agents acting on the renin-angiotensin system	5	15.6
Lipid modifying agents	5	15.6
Beta blockers	6	18.8
Other	6	18.8
Total	32	100

## Discussion

In 2013, The Joint Commission International [21] recognized that organizations faced challenges in implementing medication reconciliation. The effectiveness of this procedure requires a thorough understanding of the patient's current prescriptions and the drugs used at home. This process is part of the list of National Patient Safety Goals for the Accreditation of Hospitals, which is incorporated as target number three: “Enhancing drug use safety” [21].

Studies have been published where the pharmacist is primarily responsible for medication reconciliation [19], [22]–[23]. Kliethermes (2008) [24] concludes that the pharmacist should play an important role in this process, since he is the most qualified professional to obtain and document an accurate and complete medication history.

In contrast, Coffey et al (2009) [25] concluded that it is difficult to justify widespread implementation of clinical pharmacists to obtain the medication history due to competing duties, limited staffing resources and insufficient evidence to justify routine medication reconciliation in all patients across all settings.

Some studies have evaluated specific criteria for conducting reconciliation based on risk factors reported by patients [20], [26]–[28]. However, these criteria vary among authors. Some suggest that patients over 65 years of age or the presence of polypharmacy [20], [29], [30] are criteria indicating a need for medication reconciliation. In Buckley et al (2013) [28], the criteria for deciding which patients received medication reconciliation on admission were diagnosis of heart failure or acute myocardial infarction. Many studies [26], [30], [31] show that drugs for the treatment of cardiovascular diseases are the most likely to be involved in unintentional discrepancies. Therefore, the cardiology unit may be a service with high-risk patients for the occurrence of these discrepancies. More specific studies are needed to confirm this hypothesis.

The participation of other professionals can contribute to the effectiveness of medication reconciliation [32]. Generally, three professions are involved in obtaining the medication history – doctors, nurses and pharmacists – but there is little agreement on the role and responsibilities of each professional regarding medication reconciliation, and the procedures used lack standardization [32].

In our study, the pharmacist played a major role in achieving reconciliation with 50 of the 58 patients having at least one discrepancy. Only eight (14%) patients had their home prescriptions maintained upon admission and unintentional discrepancies were identified in half of the patients. Most of the discrepancies in our study were justified, similar to the study by Paez et al. (2010) [33], which included 469 patients and where 3,609 drugs were reconciled, of which 2,466 (68.3%) had discrepancies: 667 (27.1%) unintentional and 1,799 (72.9%) justified. Unintentional discrepancies may be considered medication errors, which may have clinical consequences, that is, they can cause harm or be potentially harmful [34].

In a systematic review of 25 medication reconciliation study by Alfaro-Lara et al (2013) [35], the authors found that most of these (16 studies) outlined drug omission as the most frequent discrepancy. Another systematic review to assess the frequency, type and clinical importance of medication history errors on admission showed that there is considerable variation in the definition of this issue. Some studies include only errors of omission, while others also include errors of dose, frequency and in the introduction of unintentional medication, thus evidencing the aforementioned variation [29].

In the present study, discrepancies in the dose, route of administration or frequency were the most common 41% (13), followed by the omission of drugs 35% (11). The study by Reeder and Mutnic (2008) [36], which compared the medication history gathered by doctors and pharmacists, showed that pharmacists documented significantly more errors in medication doses and dosage schedules than physicians (614 versus 446 and 614 versus 404, respectively, p≤0.001 for both comparisons). The authors concluded that pharmacists conducted a more comprehensive medication history. Among the flaws in the collection of medication history by physicians, the authors emphasized the lack of specific and relevant information, such as specific drugs, drug doses, dosages schedules, allergies, and vaccination status.

Most unintentional discrepancies were classified in the category error without harm, using the scale proposed by Gleason et al (2010) [20]. Similar results were found in other studies [20], [23], [37].

The discrepancies identified in this study and appropriate recommendations were verbally communicated to prescribers and most of them (74%) were accepted. Of the 32 unintentional discrepancies identified, 31 were discussed with prescribers. In the medication reconciliation study carried out by Gleason et al (2004) [37], pharmacists performed 97 interventions involving 55 patients, and most of the recommended interventions (71%) were accepted by physicians. In six cases, investigators were unable to determine whether the clinician accepted the pharmacist's suggestion or the doctor was not available to perform the intervention.

A controlled clinical trial demonstrated that reducing discrepancies, coupled with instructions to the patient and pharmacist monitoring is associated with lower rates of preventable adverse drug-related events, and a reduction of the need for emergency service care or hospital admission [38].

## Conclusions

The most important outcome in our study, was the effectiveness of pharmacists in intercepting and correcting prescribing errors before they resulted in harm. This study identified 181 discrepancies in 50 patients and identified the types of discrepancies and their potential for harm.

There are two important findings from our research: 1) we identified design flaws in how the medication history is collected and documented on admission to the cardiology ward of a University hospital; and, 2) pharmacist interception and correction of medication errors is an important safety practice to identify potential problems and prevent patient harm.

This study may help guide reconciliation activities in other cardiology clinics of similar hospitals.
