# Correction: Medication Reconciliation in Patients Hospitalized in a Cardiology Unit

**DOI:** 10.1371/journal.pone.0123362

**Published:** 2015-03-31

**Authors:** The PLOS ONE Staff

The second author’s name is spelled incorrectly. The correct name is: Gláucia Noblat de Carvalho Santos.

Additionally, the fourth author’s name is spelled incorrectly in the citation.

The complete, correct citation is:

Magalhães GF, Santos GNC, Rosa MB, Noblat LACB (2014) Medication Reconciliation in Patients Hospitalized in a Cardiology Unit. PLoS ONE 9(12): e115491. doi:10.1371/journal.pone.0115491
